# Changes in Physical Activity and Sedentary Behavior in Chinese Young Adults during the COVID-19 Pandemic: A Repeated-Measure Study throughout One Year

**DOI:** 10.3390/healthcare9111404

**Published:** 2021-10-20

**Authors:** Ke Ning, Si-Tong Chen, Xinli Chi, Kaixin Liang

**Affiliations:** 1 School of Physical Education and Sport, Shaanxi Normal University, Xi’an 710119, China; 2 Institute for Health and Sport, Victoria University, Melbourne 8001, Australia; sitong.chen@live.vu.edu.au; 3 School of Psychology, Shenzhen University, Shenzhen 518060, China; xinlichi@126.com (X.C.); liangkaixin2020@email.szu.edu.cn (K.L.)

**Keywords:** behavioral epidemiology, moderate to vigorous physical activity, light physical activity, sedentary behavior, young adults, seasonal change, quarantine

## Abstract

Though we know physical activity (PA) decreased while sedentary behavior (SB) increased compared to that before the COVID-19 pandemic, little is known about subsequent changes in PA and SB throughout one year in the post-pandemic era. This study aimed to examine the changes in PA and SB in a sample of Chinese young adults using a four-wave repeated-measure design during the pandemic. A total of 411 participants provided self-reported data of sociodemographic characteristics (e.g., sex, age), PA, and SB. Nonparametric tests and generalized estimating equations were conducted. Results revealed significant changes in moderate to vigorous PA (MVPA), light PA (LPA), and SB. The MVPA of Wave 1, 2, and 3 was significantly less than that of Wave 4 (*p* < 0.001); the LPA of Wave 1 and 2 was significantly less than that of Wave 4; the SB of Wave 1 was significantly more than that of Wave 4 (*p* < 0.05). Being female was the only predictor of changes in MVPA (Beta = −0.311, *p* *<* 0.001). Being female (Beta = 0.115, *p* = 0.003) and perceived family affluence (Beta = −0.059, *p* *<* 0.001) were predictors of changes in SB. As such, PA was less, while SB was more during the early stages of the pandemic. With the progress of the pandemic stages, health behaviors in young adults have been gradually improved. Sex and perceived family affluence were two important factors in predicting health behaviors. Our results can inform efficient policies or interventions in the COVID-19 era and future similar public health events.

## 1. Introduction

The risks of insufficient physical activity (PA) and excessive sedentary behavior (SB) on the health burden have been well documented in the previous literature [1,2,3,4,5,6,7]. For example, scientific evidence based on COVID-19 has shown that changes in PA increased the risk of weight gain and cardiovascular disease [8,9]. Besides, reduced PA was associated with a greater presence of depression and anxiety symptoms [10,11]. However, the prevalence of insufficient PA and excessive SB are high worldwide and are ongoing public health concerns [12,13]; of note, the current COVID-19 pandemic has created a circumstance that may make this situation even worse [14,15]. Since the outbreak of COVID-19, people’s lifestyles across the world have been dramatically changed, owing to the corresponding social distancing and lockdown. The measures of social distancing, effective for controlling the pandemic, also produced side effects of directly altering individuals’ movement behaviors [16,17]. Many studies reveal that the PA level adopted after the outbreak of the pandemic was lower than that adopted prior to the pandemic [10,18]. Meanwhile, SB increased and a more common sedentary lifestyle has been observed in young adults during the pandemic [14]. Further, a series of issues regarding public health have been raised, with aggravated mental and physical health problems affecting entire populations, including young adults [19,20,21]. Given the important health impacts of PA and SB [22,23,24], studies on the levels and changes of PA and SB are needed to inform health policymakers and practitioners to develop health education and behavior interventions on the specific population during this pandemic, and other future relevant events.

As a concept related to PA, but less explored, SB is characterized by low energy expenditure (≤1.5 metabolic equivalents of task) in a sitting or reclined posture during waking hours [25]. It has been noted that SB may have little association with PA and thus someone can accumulate large amounts of both PA and SB within one day [26,27]. Taken together, too much SB and too little PA represent independent and distinct risk factors for individuals’ health. Indeed, SB has also been recognized as a factor affecting individuals’ overall health during the COVID-19 pandemic [14,28], while excessive SB has also been associated with negative physical health indicators during this time [29]. Furthermore, SB imposed a negative impact on the mental health of college students during the pandemic [11,30]. As such, the practice of increased PA and reduced SB are both important for health promotion during the ongoing and would-be-long-lasting public crisis. Many studies have also concurrently explored changes in PA and SB in young adults before and during the COVID-19 pandemic [31]. For example, a review to summarize literature that investigated differences in PA and SB before vs. during COVID-19 lockdown(s) found that the majority of studies reported decreases in PA and increases in SB during their respective lockdowns, across several populations [31]. A longitudinal cohort study found that university students’ movement behaviors have been impaired during the lockdown, with the decreased PA and increased SB [21]. Besides, a cohort study conducted four times measurements between 23 April and 11 December 2020 among French university students [32]. The study found that PA levels initially increased during the first lockdown but showed a subsequent decline and followed different trajectories depending on the intensity of PA, whereas SB levels were high and tended to persist over time.

Nevertheless, while most existing longitudinal studies compared levels of PA and SB between two time points (pre and during the pandemic), few studies have adopted a more intensive longitudinal design to observe the changes in PA and SB during the pandemic. However, the pandemic has been predicted to last for a long time, suggesting that we will have to coexist with COVID-19 for a long period. In the real world, this expectation has been true, as many countries are suffering from the COVID-19, and a large number of cities or communities are switching the status between lockdowns starting and ending repeatedly. This scenario informs that previous studies focusing on changes in PA and SB at two survey points may inhibit researchers to obtain a more comprehensive understanding of changes in PA and SB during the pandemic. Tracking how PA and SB change in a longer period with more repeated measurements can help to depict a more accurate profile of movement changes, and thus can better inform behavioral changes plan for health promotion. However, to our knowledge, there is no study investigating changes in PA and SB throughout one year. Hence, the current study aims to fill the literature gaps and adds to evidence about seasonal changes in PA and SB in young adults during the COVID-19 era.

Moreover, to better understand behavior changes in young adults, sociodemographic correlates of PA and SB during the COVID-19 pandemic were needed to be identified. Demographic factors (e.g., sex) and physical characteristics (e.g., body mass index [BMI]) have been shown to be associated with changes in PA and SB during the COVID-19 pandemic [18,33]. Through the lens of the socio-ecological model, a framework for behavioral changes, researchers have proposed that intra-individual (e.g., age), inter-individual (e.g., perceived family affluence), environment (e.g., residence) determinants would potentially change behaviors of youth during the pandemic [34].

Collectively, the primary aim of this survey study was to examine between-season variation in PA (MVPA and LPA) and SB in a sample of Chinese university students during the pandemic period; additionally, this study aimed to examine sociodemographic correlates (age, sex, BMI, perceived family affluence, and residence) of PA and SB during the COVID-19 pandemic. The results of this study may assist in designing efficient strategies to cope with healthy movement behaviors during the present and next quarantine periods.

## 2. Methods

### 2.1. Study Survey and Participants

This study used data from a four-wave one-year longitudinal study conducted during the pandemic (surveyed at a three-month interval), aiming to understand changes in lifestyle behaviors and mental health among Chinese university students in the context of the COVID-19 pandemic. Initially, a convenient sampling method was taken in the current study. Study participants were mainly recruited using an online social network, by positing our research protocol through social media platforms (e.g., WeChat, Weibo, and QQ). The inclusion criteria were to be a college student in China, willing to participate in the study and be able to communicate in Chinese. Participants who provided consent to be included in our research were sent a survey link to an online questionnaire that required around 20 min to complete. In Wave 1, 1365 Chinese college students participated in our survey.

The brief information of the four waves involved is as follows: Wave 1 (13–22 May 2020): most Chinese students were subject to confinement due to COVID-19; Wave 2 (21–31 August 2020): most college students had stayed at home for more than six months; Wave 3 (20–25 November 2020): students had returned to campus with a normal academic and social life to a large extent; Wave 4 (25 February–1 March 2021): students were at the end of the winter vacation and are ready for new semester. Finally, a total of 411 study participants completed all four waves, providing data on all the variables this study needed for analysis (including sex, age, BMI, residence, perceived family affluence, PA, and SB). The 411 study participants composed the final analytical study samples. The sample was calculated using G*Power for repeated measures analysis with the following assumptions: an expected small effect size (*f* = 0.10), a margin of error of 5%, and power of 80%. The calculated required sample size is 222. The final sample (*n* = 411) goes beyond this, which is sufficient to meet the excepted statistical requirements. Study participants’ recruitment and data collection procedures, as well as details of research protocol, were informally approved by the Human Research Ethics Board (No: 2020005) at Shenzhen University. More details of the study survey methodology can be found elsewhere [35].

### 2.2. Physical Activity and Sedentary Behavior

PA and SB were assessed by the International Physical Activity Questionnaire Short Form (IPAQ-SF). Participants were asked to report their time spent in sitting (SB), walking (LPA), and moderate and vigorous PA (MVPA) over the past seven days. The IPAQ-SF has been validated with acceptable validity and reliability in the Chinese adult population in previous studies [36,37]. In detail, the IPAQ-SF had an intraclass correlation coefficient (ICC) of 0.97 (95% confidence interval [CI]: 0.95–0.98) for SB, an ICC of 0.85 (95% CI: 0.75–0.91) for moderate PA, and 0.75 (95% CI: 0.60–0.85) for vigorous PA, showing being reliable in assessing PA and SB in Chinese adult population [3]. For validity, previous research also showed acceptable validity of the IPAQ-SF against device-based measures of PA [37].

### 2.3. Socioeconomic Demographics

Some socioeconomic demographic characteristics were assessed using an online self-reported questionnaire, including sex (male or female), age, residence location (urban or rural), height (cm), and weight (kg). In addition to these, perceived family affluence was assessed by a scale (0–10; with higher scores representing better perceived family affluence), which has shown acceptable reliability and validity in the Chinese population [38,39].

### 2.4. Statistical Analysis

The Shapiro–Wilk test examined the distribution of the raw data, which confirmed that all data were not normally distributed. Hence, statistical analysis based on normal distribution could not be applied in our study when doing advanced analysis in addition to descriptive statistics. Descriptive statistics were used to report sample characteristics, levels of MVPA, LPA, and SB (means and standard deviation [SD]) at different survey time points. To examine the sex difference in age, BMI, and perceived family affluence, Mann-Whitney U test was used to achieve the related aims; then, a Chi-square test was performed to test sex differences in residence location. A nonparametric test (Kruskal-Wallis) with repeated measures was used to examine the changes in MVPA, LPA, and SB. In this regard, the Durbin-Conover post-hoc test was used to report the differences in MVPA, LPA, and SB at different survey time points. To examine if sociodemographic factors had effects on these behaviors, sex, residence, age, BMI, and perceived family affluence were entered as fixed factors. Generalized Estimating Equations (GEE) with a Gamma Positive Link function were used to analyze the changes in MVPA, LPA, and SB during the COVID-19 pandemic and their associated predictors (based on control variables). The level of significance was set at *p* < 0.05 and analyses were done using Statistical Package for Social Science Version 26.0 (IBM Corp, Armonk, NY, USA).

## 3. Results

Table 1 details sample characteristics in this study. Of the 411 included study samples, males accounted for 30.9%, while females accounted for 69.1%. The mean age of the study sample was 20.6 with a standard deviation [SD] of 1.8. There was no sex difference in age (*p* = 0.489). The mean BMI was 21.8 (SD = 6.6), with a statistically significant sex difference (*p* *<* 0.001). As for perceived family affluence, the mean of the study sample was 4.9 (SD = 1.3) without sex difference (*p* = 0.130). For residence, more than 50% of study samples lived in urban areas and more females lived in urban areas than males (*p* = 0.027). 

Table 2 provides the descriptive characteristics of MPVA, LPA, and SB at different survey time points. The means (SD) of MVPA were 15.63 (25.61), 19.11 (30.36), 13.92 (24.06), 28.17 (40.35), respectively. The means (SD) of LPA were 40.96 (50.67), 34.59 (34.67), 50.49 (38.30), and 50.48 (38.94), respectively. The means (SD) of SB were 449.5 (200.96), 429.78 (230.76), 410.07 (219.85), and 414.68 (214.23), respectively. More details on MVPA, LPA, and SB can be found in Table 2.

**Figure 1:**
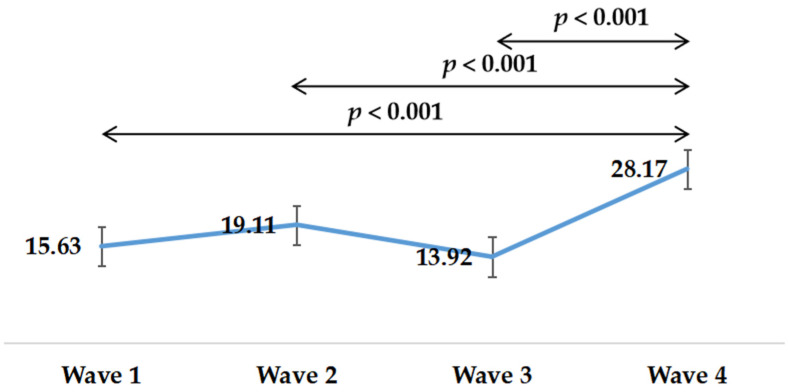
Changes in moderato to vigorous physical activity in four waves during the COVID-19 pandemic.

Figure 1 presents changes in MVPA during the survey period. MVPA time presented an upward trend. MVPA time of Wave 1 (*p* < 0.001), Wave 2 (*p* < 0.001), and Wave 3 (*p* < 0.001) were significantly less than that of Wave 4.

**Figure 2:**
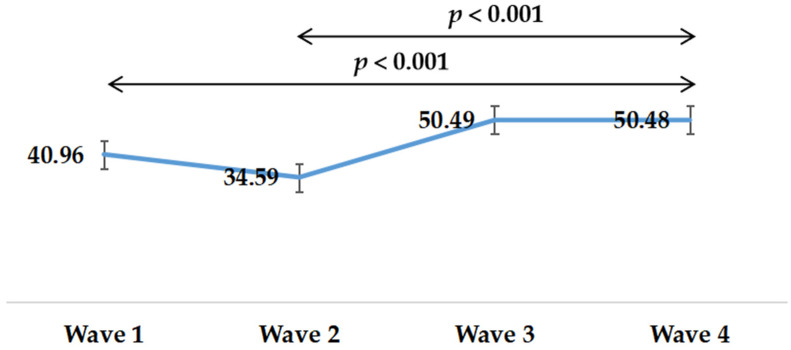
Changes in light physical activity in four waves during the COVID-19 pandemic.

Changes in LPA during the survey period were shown in Figure 2. LPA time presented an upward trend. LPA time of Wave 1 (*p* < 0.001) and Wave 2 (*p* < 0.001) was significantly less than that of Wave 4.

**Figure 3:**
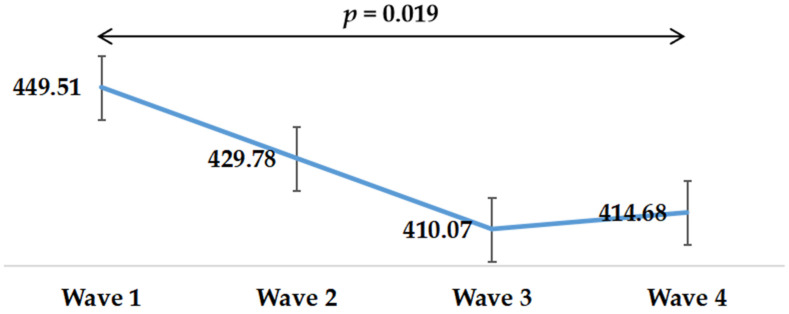
Changes in sedentary behavior in four waves during the COVID-19 pandemic.

Changes in SB during the survey period can be observed in Figure 3. SB time presented a downward trend. SB time in Wave 1 was significantly more than that of Wave 4 (*p* = 0.019).

Table 3, Table 4 and Table 5 presents the results from GEE on the predictors of changes in MVPA, LPA, and SB of the study participants. In our results, residence, age, perceived family affluence, and BMI were not predictors of changes in MVPA in study samples, while sex was the only predictor of changes in MVPA in study samples, indicating that female young adults were more likely to report less time spent in MVPA compared with their male counterparts (Table 3). As for LPA, there were no significant associations between selected factors and outcomes, suggesting that no predictors of LPA in our study (Table 4). Concerning SB, it was evident that sex and perceived family affluence were significant predictors of changes in SB in study samples (Table 5).

## 4. Discussion

Prior research on the differences in movement behaviors between before and after the COVID-19 pandemic has revealed PA decreased while SB increased in different countries or regions because of quarantine caused by the COVID-19 [10,40,41]. With a more intensive longitudinal design, we add information to the changes of PA and SB from the early lockdown stages of the COVID-19 pandemic to the subsequent time [41]. Our findings may fill the research gaps and provide meaningful evidence on the impacts of COVID-19 on people’s health behaviors. We found that compared to the later remission stage of the pandemic, both MVPA and LPA were significantly higher in the early stages, especially PA. Concerning the predictors of changes in PA and SB, we found that sex was a significant predictor of changes in MVPA while sex and perceived family affluence were predictors of changes in SB; no significant predictors were found for LPA in this study. More detailed discussions are presented below.

Our study found that PA (MVPA and LPA) in the early stages were significantly lower and SB was significantly higher than that in the later remission period. Echoing that research compared the levels of PA or SB between during and before the pandemic, findings of this study also revealed the negative impact of COVID-19 on individuals’ health by observing less PA and more SB observed in the early pandemic stage. Numerous studies have suggested that the COVID-19 has led to impacts on people’s health behaviors, especially on movement-related activities (PA, SB, and sleep) [31,42,43]. These considerably significant changes make people’s daily life unhealthy, namely, as insufficient PA and excessive SB can lead to many health risks for people. Of note, the results of this study could also be interpreted in a more positive perspective: with the progress of pandemic stages, PA (MVPA and LPA) presented an upward trend, while SB presented a downward trend. This research finding may be interpreted that as restrictions gradually conceal, young adults have larger space to engage in PA, such as active travel outdoors. Another possible reason for the increased PA is that Chinese college students were able to return to campus in batches for a normal life as prevention and control measures in China have been effective in curbing the expansion of the COVID-19. Another finding is that in the later seasons during the pandemic, reduced SB among young adults was observed. After the relevant restrictions against COVID-19 were revoked, SB would decrease as PA increased, because the time spent in SB might be replaced by PA. In addition, when people did not have to stay at home to study or work, the relevant screen use time could be reduced, which led to a further reduction of overall SB [31]. In other words, this result implies that despite COVID-19 being an ongoing situation, individuals could gradually adapt to the situation and return to a normal lifestyle.

When looking at predictors of changes in PA and SB, some interesting findings are worthy of further interpretation. As for MVPA, compared with males, females were more likely to report less time spent in MVPA. This finding was consistent with much previous relevant literature concerning health behaviors during the pandemic [18,44,45,46,47,48]. However, some other studies negated our finding that sex was not an important predictor of MVPA during the pandemic; for example, Rhodes et al., found that there were more important predictors explaining the changes in MVPA [40]. The discrepancy may be owing to more potential predictors included in Rhodes et al.’s study, which in turn reduces the explaining variance of sex in changes in MVPA. No matter the inconsistency between our study and the other studies, the current study also adds to evidence that the socio-ecological model (SEM) can be also applied to explain health behaviors during this special situation, though SEM has been a well-recognized theoretical framework to explain factors of MVPA in many contexts without a pandemic. Possible reasons to explain why female young adults’ lower levels of MVPA include males’ stronger awareness of engaging in or maintaining MVPA than females, and preference to make use of available discretionary time to do more MVPA. On the contrary, our study did not find any predictors of LPA, but this cannot indicate that SEM cannot explain LPA, because the evidence on this in the literature remains lacking. Thus, it is encouraged that more studies can explore the predictors of LPA in young adults during the pandemic. Compared with MVPA, LPA is a relatively easy-performed kind of activity, as it requires lower energy expenditure, and it can occur more frequently without demographic differences. In this regard, it seems reasonable that no predictor would affect LPA in young adults during the pandemic.

This study found that sex and perceived family affluence were predictors of changes in SB in young adults, a research finding that is consistent with some previous studies [14,33,49]. Compared with male young adults, female counterparts were more likely to report more time spent in SB, which can be supported by previous studies [50,51]. A possible reason to explain this research finding is that during the pandemic, young male adults were more likely to find approaches to engage in PA for health promotion [52,53] when academic loads and social activities were fewer during the pandemic. Unlike the association between sex and SB, perceived family affluence was a negative predictor of SB in young adults, which indicates higher family affluence and less SB. This might be because young adults from families with a higher family affluence have a better awareness of the adverse effects of excessive SB [54,55]. However, this interpretation is based on previous studies conducted prior to COVID-19, which thus need more contextual evidence to support our assumption. In this regard, more studies, especially using longitudinal research design, are encouraged to explore the predictors of changes in SB in populations. Such evidence is beneficial to health promotion during the unique period or future similar public events.

The obvious strength of this study was using an intensive longitudinal design with four repeated measurements. On the basis of previous relevant studies that have investigated differences of PA and SB between before and during the COVID-19 pandemic, our study, in another perspective, further verified the negative impacts of the pandemic on people’s health behaviors. Further, we measured MVPA, LPA, and SB concurrently, which is another advantage of this study compared to other studies. However, some study limitations must be acknowledged. One of the limitations is that the sample size was relatively small (<500), which may reduce the generalizability of research findings. Second, the sampling technique this study used was not a random sampling, and accordingly, the representativeness of the sample was restricted. Third, owing to the social distancing caused by COVID-19, device-based assessments cannot be achieved, and the current study used a self-reported questionnaire to collect data. Fourth, compared with some studies that investigate the levels of PA and SB before COVID-19, our study merely had data on PA and SB during the pandemic, which limited a comprehensive understanding of changes in PA and SB. Future studies are encouraged to consider longer tracks of PA and SB in young adults from pre-lockdowns to later periods, which can produce more insightful information for public health promotion. Fifth, all of our study participants came from Guangdong Province (Southern China); thus, research findings based on our study could not be extended into other regions with different cultural, socioeconomic, and societal characteristics. Moreover, concerning the predictors of PA and SB, owing to limited availability of the measures, only some simple sociodemographic factors (e.g., sex, age, BMI) were considered in our analysis, which may inhibit our understanding of correlates of PA and SB in young adults during the pandemic. Finally, in our study, the distribution of males (about one-third) and females (about two-thirds) was not equal. Future studies are encouraged to address these limitations.

## 5. Conclusions

This study investigated the changes in PA (MVPA and LPA) and SB during the COVID-19 pandemic and further provides evidence of the impacts of the pandemic on populations using a sample of Chinese college students. Moreover, as seen during the studied year of the COVID-19 pandemic, in order to promote PA in young adults for health promotion, it is necessary to pay attention to female young adults; while targeting female young adults and those with a lower family affluence can be helpful in reducing excessive SB during quarantine.

## Figures and Tables

**Table 1:** Sociodemographic characteristics at baseline of the study sample.

Variable	Total (*n* = 411)	Male (*n* = 127, 30.9%)	Female (*n* = 284, 69.1%)	*p*
Age	20.6 (1.8)	20.5 (1.7)	20.7 (1.8)	0.489
Body mass index	21.8 (6.6)	23.3 (7.6)	21.1 (6.0)	<0.001
Perceived family affluence	4.9 (1.3)	4.8 (1.3)	5.0 (1.3)	0.130
Residence				
Urban	237 (57.7%)	63 (49.6%)	174 (61.3%)	0.027
Rural	174 (42.3%)	64 (50.4%)	110 (38.7%)	

Note. Data are shown as mean (standard deviation) or *n* (percentage).

**Table 2:** Descriptive characteristics of physical activity and sedentary behavior in different waves.

Variable	Wave 1		Wave 2		Wave 3		Wave 4	
	Mean (SD)	Median (Range)	Mean (SD)	Median (Range)	Mean (SD)	Median (Range)	Mean (SD)	Median (Range)
MVPA	15.63 (25.61)	7.63 (0.00–160.30)	19.11 (30.36)	8.66 (0.00–169.30)	13.92 (24.06)	6.54 (0.00–177.60)	28.17 (40.35)	13.48 (0.00–169.00)
LPA	40.96 (50.67)	21.43 (0.00–180.00)	34.59 (34.67)	25.71 (0.00–180.00)	50.49 (38.30)	42.86 (0.00–180.00)	50.48 (38.94)	40.00 (0.00–180.00)
SB	449.51 (200.96)	466 (249.50–850.60)	429.78 (230.76)	421.00 (195.30–824.60)	410.07 (219.85)	420.00 (210.70–848.30)	414.68 (214.23)	420.00 (200.20–818.30)

Note. MVPA: moderate to vigorous physical activity; LPA: light physical activity; SB: sedentary behavior. Data are shown with the unit of min/day.

**Table 3:** Predictors of changes in moderate to vigorous physical activity during the COVID-19 pandemic.

Variable	Beta	Std. Error	95% Wald Confidence Interval		Chi-Square	*p*
Sex						
Female	−0.311	0.077	−0.462	−0.160	16.379	<0.001
Male	REF					
Age	−0.022	0.019	−0.06	0.016	1.306	0.253
Body mass index	0.005	0.005	−0.005	0.015	1.052	0.305
Perceived family affluence	−0.002	0.03	−0.061	0.057	0.006	0.937
Residence						
Rural	0.019	0.073	−0.124	0.161	0.065	0.799
Urban	REF					

Note. REF: reference group.

**Table 4:** Predictors of changes in light physical activity during the COVID-19 pandemic.

Variable	Beta	Std. Error	95% Wald Confidence Interval		Chi-Square	*p*
Sex						
Female	−0.017	0.0596	−0.134	0.100	0.084	0.772
Male	REF					
Age	−0.023	0.0157	−0.054	0.008	2.126	0.145
Body mass index	0.005	0.0038	−0.003	0.012	1.672	0.196
Perceived family affluence	0.000	0.0205	−0.041	0.040	0.000	0.986
Residence						
Rural	0.053	0.0534	−0.052	0.158	0.990	0.32
Urban	REF					

Note. REF: reference group.

**Table 5:** Predictors of changes in sedentary behavior during the COVID-19 pandemic.

Variable	Beta	Std. Error	95% Wald Confidence Interval		Chi-Square	*p*
Sex						
Female	0.115	0.038	0.041	0.19	9.133	0.003
Male	REF					
Age	−0.013	0.009	−0.031	0.005	1.913	0.167
Body mass index	−0.006	0.002	−0.010	−0.001	5.931	0.015
Perceived family affluence	−0.059	0.014	−0.086	−0.032	18.621	<0.001
Residence						
Rural	−0.055	0.034	−0.121	0.012	2.583	0.108
Urban	REF					

Note: REF: reference group.

## Data Availability

The data analyzed in this study are available from the authors on reasonable request.
